# Effect of Rare Earth Ce on Microstructure and Properties of Q370qENHY Bridge Steel

**DOI:** 10.3390/ma18051048

**Published:** 2025-02-27

**Authors:** Yue Chen, Jichun Yang, Xiangjun Liu

**Affiliations:** 1 School of Rare Earth Industry, Inner Mongolia University of Science and Technology, Baotou 014010, China; chenyue14747350223@163.com; 2 Key Laboratory of Green Extraction & Efficient Utilization of Light Rare-Earth Resources, Ministry of Education, Inner Mongolia University of Science and Technology, Baotou 014010, China

**Keywords:** rare earth, microstructure, mechanical properties, corrosion resistance

## Abstract

To enhance the mechanical characteristics and corrosion resistance of bridge steel, three distinct groups of test steels with varying Ce contents were formulated. The objective was to investigate the influence of rare earth Ce on the microstructure, impact performance, and corrosion resistance of bridge steel. The addition of rare earth elements improves both the impact performance and the corrosion resistance of bridge steels. The present research systematically examines the impact of cerium (Ce) incorporation on the structural and impact performance of bridge construction steels, with particular emphasis on elucidating the fundamental mechanisms governing these modifications. This investigation establishes a comprehensive theoretical framework that facilitates the advancement of next-generation rare earth-enhanced high-performance steel alloys specifically designed for bridge engineering applications. The investigation reveals that rare-earth elements exert a significant influence on microstructural refinement, leading to the diminution of grain size. Additionally, these elements catalyze the modification of inclusion morphology in the test steel, transitioning from an irregular form to a spherical one, with a concomitant decrease in inclusion size. The tested steel with a rare earth mass fraction of 0.0025 wt.% has the best impact performance and the lowest corrosion rate. The impact performance improved by 7.37% compared with the experimental steel without the addition of rare earth elements. The incorporation of rare earth elements has been observed to promote the accumulation of Cu in the rust layer, which contributes to the improved stability of the layer. Concurrently, it has been noted that, for equivalent periods of corrosion exposure, there is a positive correlation between the arc radius of bulk resistance and the incremental levels of rare earth Ce.

## 1. Introduction

With the rapid development of infrastructure in China, particularly the increasing demand for high-speed rail, cross-river and cross-sea bridges, as well as urban bridge projects, the performance and quality requirements for bridge steels have become more stringent. Traditional bridge steels are no longer adequate to meet the demands of modern bridge construction, which requires high strength, large spans, and lightweight structures. As a result, they have gradually been replaced by bridge steels that offer superior strength, toughness, and corrosion resistance [1]. There are various types of weathering steels used in bridge construction, such as the HPS, A572, and A709, developed in the United States, all of which exhibit commendable mechanical properties and excellent weathering resistance [2,3,4]. Optimizing the impact performance and corrosion resistance of steel through precise control of alloy element additions and their proportions has emerged as a central focus in the ongoing research on bridge steels. Zhang and Sun found that the addition of La and Cr can improve the corrosion resistance of steel, while Hara, Tranchida, and Fan, among others, investigated the corrosion mechanisms of rust layers and discovered that different elements influence the composition of the rust layer, thereby affecting the corrosion resistance [5,6,7,8,9,10]. Jiang [11] discovered that the addition of vanadium and nitrogen notably decreased the average size of network ferrite while simultaneously increasing the volume fraction of intragranular ferrite and ferrite, resulting in improved mechanical properties and microstructural characteristics of the steel. Zhou [12] found that niobium, when used as a microalloying element, can notably improve the hot-rolling performance of bridge steels. During the hot-rolling process, it demonstrated excellent workability and superior overall mechanical properties, particularly in terms of fatigue resistance and corrosion resistance. Wang [13] observed that titanium substantially improved the yield and tensile strength of the steel by promoting grain refinement and precipitation hardening. Wang et al. [14,15] demonstrated the significant role of chromium in welded structures, noting its ability to improve both the strength and hardenability of welds, as well as its positive influence on the corrosion resistance of steel. Kimura [16] found that weathering steel containing 3% (by weight) nickel exhibited superior corrosion resistance compared with conventional weathering steel. This improvement was attributed to the formation of Fe_2_NiO_4_ within the rust layer, which enhanced its density.

Microalloying, as a conventional approach, requires precise control over the alloying element content and smelting processes during actual production. This not only complicates the manufacturing procedures but also increases production costs. Given the abundance of rare earth resources in China, rare earth elements are considered potential alternatives to certain precious metals. These elements have the potential to enhance the corrosion resistance of bridge steels without compromising their mechanical properties. Consequently, a considerable number of researchers have undertaken comprehensive studies on the impact of various rare earth elements across different steel types. Wang [17] observed that the addition of a specific amount of rare earth elements increases the austenite recrystallization temperature, which holds significant implications for the microstructure and performance control of hot-rolled steel strips. Yan [18] found that rare earth elements contribute to the production of high-quality cast billets through the continuous casting and rolling process. Rare earth elements play a crucial role in mitigating the segregation of sulfur and phosphorus at the grain boundaries in steel while also improving the inclusion morphology and refining their size, resulting in a notable enhancement of the steel’s corrosion resistance. Peng found that Sc can alter the grain structure of materials, Wollants discovered that alloying elements can influence the microstructure of materials, thereby affecting their strength and ductility, and Song observed that Ce enhances the toughness of steel and improves its overall mechanical properties by modifying the grain boundary structure [19,20,21,22]. Opiela, Adabavazeh, and Liu et al. found that rare earth elements contribute to modifying the morphology of non-metallic inclusions, thereby helping to reduce the adverse effects of large or irregularly shaped inclusions on the impact performance of steel [23,24,25].

The present research systematically examines the influence of cerium (Ce) incorporation on the microstructural evolution and impact characteristics of Q370qENHY structural steel for bridge applications. Through comprehensive analysis of the fundamental principles governing Ce-induced modifications, this investigation establishes a scientific framework for the design and optimization of next-generation rare earth-enhanced high-performance bridge steels.

## 2. Materials and Methods

The steel selected for this study is Q370qENHY bridge steel, which was melted using a 25 kg vacuum induction furnace. The raw materials used in the smelting process, including carbon blocks, polycrystalline silicon, electrolytic manganese, niobium particles, titanium particles, copper blocks, molybdenum blocks, nickel blocks, and aluminum particles, are all of high purity. The recovery rate of the rare earth element cerium is estimated to be 30%. After the continuous casting of steel billets and their subsequent solidification and cooling, the samples are heated and held in a controlled atmosphere muffle furnace to achieve the desired metallurgical properties. The treatment temperature is set at 1200 °C, with a holding time of 2 h, and the heating rate is controlled at 5 °C per minute. Following the completion of heat treatment, the ingots are removed and sent to the rolling mill for continuous rolling, ultimately producing 30 mm steel billets. The chemical composition of the steel used in the experiment is provided in Table 1. The experimental material designated as 1# represents a rare earth-free bridge steel variant, whereas specimens 2# and 3# constitute modified bridge steel compositions containing differential concentrations of rare earth elements. The 0# composition is referenced according to the standard steel composition provided in the Chinese National Standard GB/T 714-2015.

The microstructure of the specimens was examined using an Axio Vert.A1 optical microscope (Zeiss, Jena, Germany). Prior to testing, the samples were etched in a 4% nitric alcohol solution for a duration of 2 to 3 s. Thermodynamic simulations were performed utilizing FactSage software (Software version 8.2) to predict the sequential precipitation behavior of non-metallic inclusions. The microstructural characterization of inclusions in the processed samples was conducted using an ultra-high resolution field emission scanning electron microscope (FE-SEM, Sigma 300 model, Oberkochen, Germany) and an energy dispersive X-ray spectrometer (EDS, JXA-iPH200F, Shanghai, China), in conjunction with chemical composition analysis via a direct-reading spectrometer (Labspark 1000 model, Wuxi, Jiangsu Province, China), enabling a detailed analysis of both morphology and composition. The impact performance of the specimen was evaluated using a pendulum impact testing machine. The corrosion environment was simulated using an LRHS-270-RY salt spray chamber (Shanghai, China), and the corrosion rate of the specimens was calculated. The rust layer cross-section of the specimens was examined and analyzed using a JXA-iPH200F field emission electron probe (Tokyo, Japan) with EPMA (Electron Probe Microanalysis). Electrochemical testing of the specimens was conducted using a Gamry electrochemical workstation (Warminster, PA, USA).

## 3. Results

### 3.1. Microstructural Analysis

**Figure 1:**
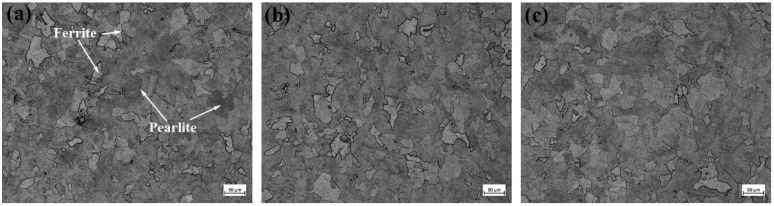
Microstructure in test steel, (**a**) 1# test steel, (**b**) 2# test steel, (**c**) 3# test steel.

Figure 1 presents the microstructures of the experimental steels containing three distinct Ce concentrations. As observed in the figure, the microstructures of the three steel groups consist of ferrite and pearlite, with the polygonal, bright white grains representing ferrite and the alternating black-and-white regions corresponding to pearlite. These two phases are distributed at regular intervals. Specifically, as shown in Figure 1a, the 1# experimental steel without Ce addition exhibits a relatively low pearlite content with an uneven distribution. The ferrite phase is more abundant, and its grain size is relatively large. In contrast, Figure 1b illustrates the 2# experimental steel, where the addition of Ce results in a reduction in both ferrite and pearlite grain sizes. The rare earth elements facilitate a slight grain refinement, and the proportion of pearlite has increased. Figure 1c further illustrates that the grain size of the 3# experimental steel is finer than that of the 2# steel. Additionally, the amount of pearlite is significantly increased, with its distribution becoming more uniform and dispersed.

**Figure 2:**
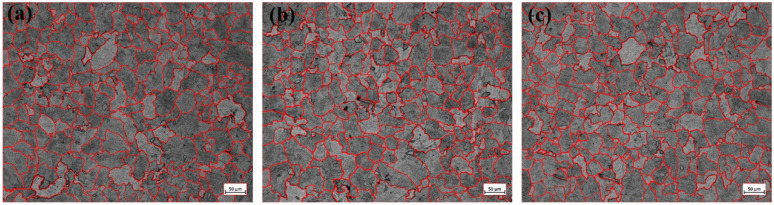
Tissue morphology with different Ce contents, (**a**) 1# test steel, (**b**) 2# test steel, (**c**) 3# test steel.

Grain size measurements of the three experimental steel groups were conducted using ImageJ software (Version 1.52), and their corresponding data are presented in Figure 2, with the summarized results provided in Table 2. Specifically, the average grain size of the 1# experimental steel is 62.4 μm, while for the 2# experimental steel, it is 57.3 μm, and for the 3# experimental steel, it is 55.7 μm. When compared with the 1# experimental steel, the 2# experimental steel exhibits an 8.17% decrease in average grain size, whereas the 3# experimental steel shows a 10.73% reduction in grain size.

Therefore, rare earth elements play a crucial role in promoting grain refinement and enhancing the distribution of pearlite, making it more uniform and dispersed. Compared with the 1# and 2# experimental steels, the 3# experimental steel exhibits the smallest average grain size. This distinctive feature substantially impedes the mobility of dislocations while restricting the advancement of crack formation, consequently promoting the structural integrity of the oxide layer formed on steel substrates. Such microstructural modifications contribute to the simultaneous enhancement of both impact performance and corrosion durability in the material system.

### 3.2. Thermodynamic Calculations of Inclusions

**Figure 3:**
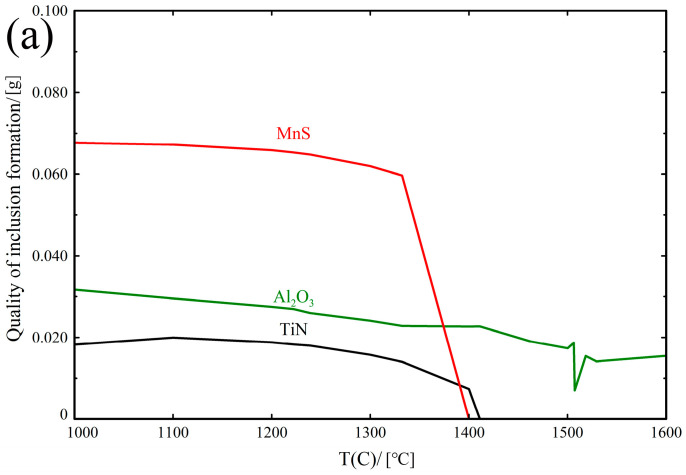

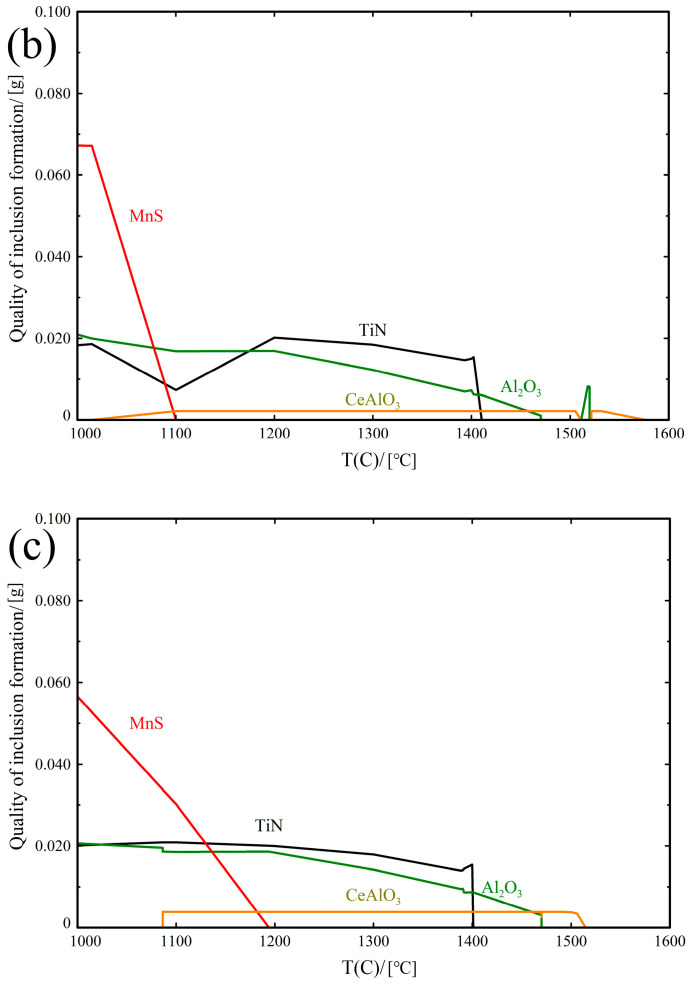
Effect of different Ce contents on the generation of inclusions, (**a**) 1# test steel, (**b**) 2# test steel, (**c**) 3# test steel.

Based on the composition of the experimental steels and calculations performed using the FactSage thermodynamic software, the sequence of inclusion precipitation is illustrated in Figure 3. Figure 3a presents the thermodynamic calculation results for the 1# experimental steel, which does not contain any rare earth elements. It can be observed that the primary inclusions in the 1# experimental steel are Al_2_O_3_, TiN, and MnS, with the precipitation of TiN and MnS gradually increasing as the temperature decreases. As the temperature drops below 1400 °C, the precipitation of TiN stabilizes, while the precipitation of MnS becomes stable when the temperature falls below 1350 °C. Al_2_O_3_, on the other hand, remains continuously stable throughout the entire temperature range in the molten steel. The sequence of inclusion precipitation in the 1# experimental steel is thus Al_2_O_3_ > TiN > MnS. Figure 3b,c present the thermodynamic calculation results for the 2# and 3# experimental steels, which contain rare earth elements. From Figure 3b,c, it can be observed that the primary inclusions in the 2# and 3# experimental steels consist of Al_2_O_3_, TiN, MnS, and CeAlO_3_. As the temperature decreases, the precipitation of TiN and MnS gradually increases. Based on thermodynamic calculations, the sequence of inclusion precipitation in the molten steel of the 2# and 3# experimental steels follows the order: CeAlO_3_ > Al_2_O_3_ > TiN > MnS. The curve of TiN in Figure 3b decreases starting from 1200 °C and then exhibits a subsequent increase at 1100 °C. This behavior can be attributed to the addition of a small amount of Ce, which influences the chemical interactions between Ti and N. Ce may either form stable compounds or alter the stability of TiN at high temperatures, resulting in a shift in the phase behavior of TiN at different temperatures.

According to the results of the thermodynamic calculations, the composition of the experimental steels, and the melting temperature, as well as the differences in the affinity of various elements for oxygen, it can be inferred that the rare earth elements, because of their relatively low concentration and strong affinity for oxygen and aluminum, preferentially react with oxygen and aluminum to form CeAlO_3_ rare earth inclusions. Based on the thermodynamic analysis of the inclusion precipitation sequence, CeAlO_3_, Al_2_O_3_, and TiN precipitate first in the molten steel. Additionally, the composite inclusions in the steel will nucleate around CeAlO_3_, Al_2_O_3_, and TiN as the core.

### 3.3. Analysis of Inclusion Types

**Figure 4:**
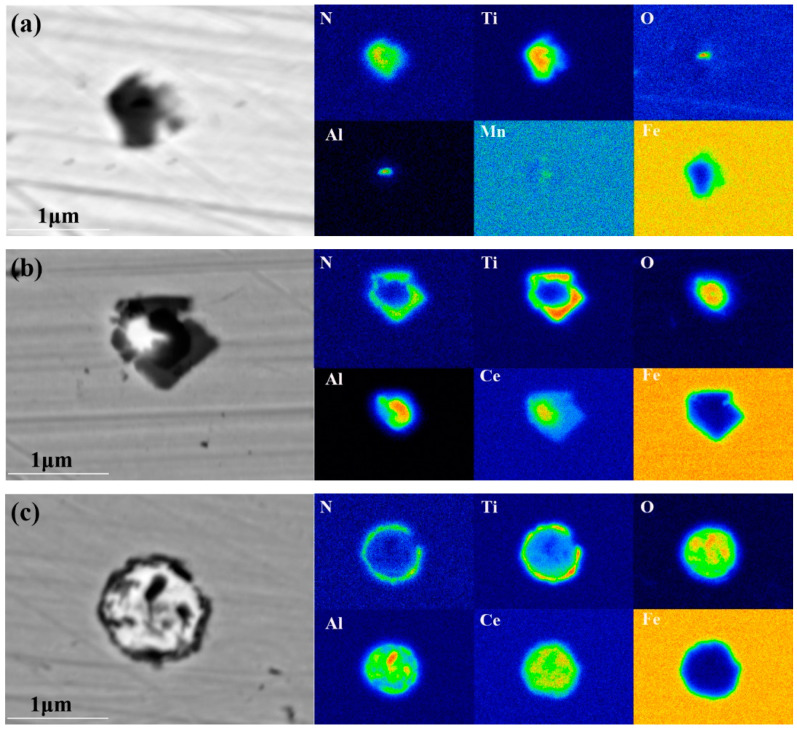
Morphology and composition of typical inclusions in the test steel, (**a**) 1# test steel, (**b**) 2# test steel, (**c**) 3# test steel.

Microstructural characterization was systematically performed to investigate the morphological features and chemical constituents of non-metallic inclusions across the three distinct steel specimens, with representative micrographs presented in Figure 4. The analytical investigations were conducted employing scanning electron microscopy (SEM) coupled with energy-dispersive X-ray spectroscopy (EDS) for comprehensive phase identification and elemental quantification. The following conclusions can be drawn from the observations. As shown in Figure 4a, the typical inclusions in the 1# experimental steel, which does not contain rare earth elements, are primarily composed of nitrogen (N), oxygen (O), aluminum (Al), and titanium (Ti). These inclusions are composite Al_2_O_3_ + TiN particles with an approximate size of 1.3 μm. The dark black regions correspond to Al_2_O_3_, while the lighter black regions surrounding the core represent TiN. Figure 4b,c present the inclusions in the 2# and 3# experimental steels, which contain rare earth elements. The inclusions are rare earth composite CeAlO_3_ + TiN particles composed of N, O, Al, Ti, and Ce. The size of these inclusions decreases with increasing rare earth content, measuring approximately 1 μm. Furthermore, it was observed that the inclusions in the 2# experimental steel exhibit irregular shapes, whereas those in the 3# experimental steel demonstrate a more uniform, spherical morphology. Based on the analysis of thermodynamic calculations, cerium (Ce) exhibits a strong affinity for oxygen (O) and aluminum (Al), leading to the preferential formation of CeAlO_3_. During the initial stages of steel melting, CeAlO_3_ precipitates first, followed by the precipitation of TiN. Ultimately, composite inclusions are formed, with CeAlO_3_ acting as the nucleation core and TiN encapsulating the outer layer.

The incorporation of cerium (Ce), a strategically selected rare earth element, induces substantial modifications in the morphological characteristics of steel inclusions. This metallurgical refinement process facilitates the transition of inclusion geometry from angular, irregular configurations to well-defined spherical morphologies, accompanied by a marked decrease in dimensional parameters, particularly in terms of average inclusion diameter. Furthermore, the incorporation of the rare earth element cerium (Ce) alters the composition of the inclusions, transforming them from the original Al_2_O_3_ + TiN into rare earth composite inclusions of CeAlO_3_ + TiN in the experimental steels.

### 3.4. Impact Performance Test

**Figure 5:**
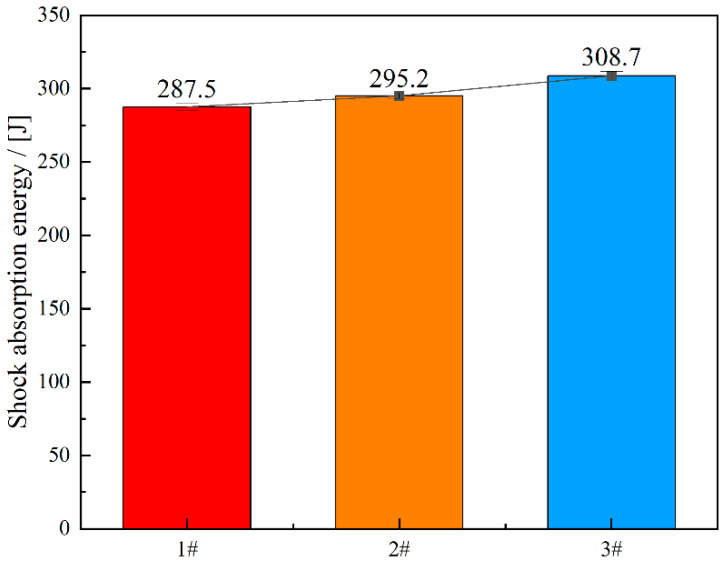
The impact absorption energy of the test steel with different Ce contents.

Figure 5 presents the impact test results at room temperature for experimental steels with varying cerium (Ce) content, with these data representing the average values obtained from three trials. The test specimens are impact samples with a V-shaped notch. The energy absorption characteristics during impact deformation, as illustrated in Figure 5, demonstrate significant variations among the investigated specimens. Specifically, the measured impact absorption energies were determined to be 287.5 J, 295.2 J, and 308.7 J for specimens designated as 1, 2, and 3, respectively. This systematic increase in energy absorption capacity across the specimen series suggests a progressive enhancement in impact resistance properties, potentially attributable to microstructural modifications or compositional variations implemented during material processing. The experimental results indicate that, with an increase in the cerium (Ce) content, a noticeable enhancement in the impact absorption energy is observed across all three groups of experimental steels. Specifically, the impact absorption energy of Sample 2 increased by 2.68% compared with Sample 1, while Sample 3 exhibited a 7.37% improvement over Sample 1.

**Figure 6:**
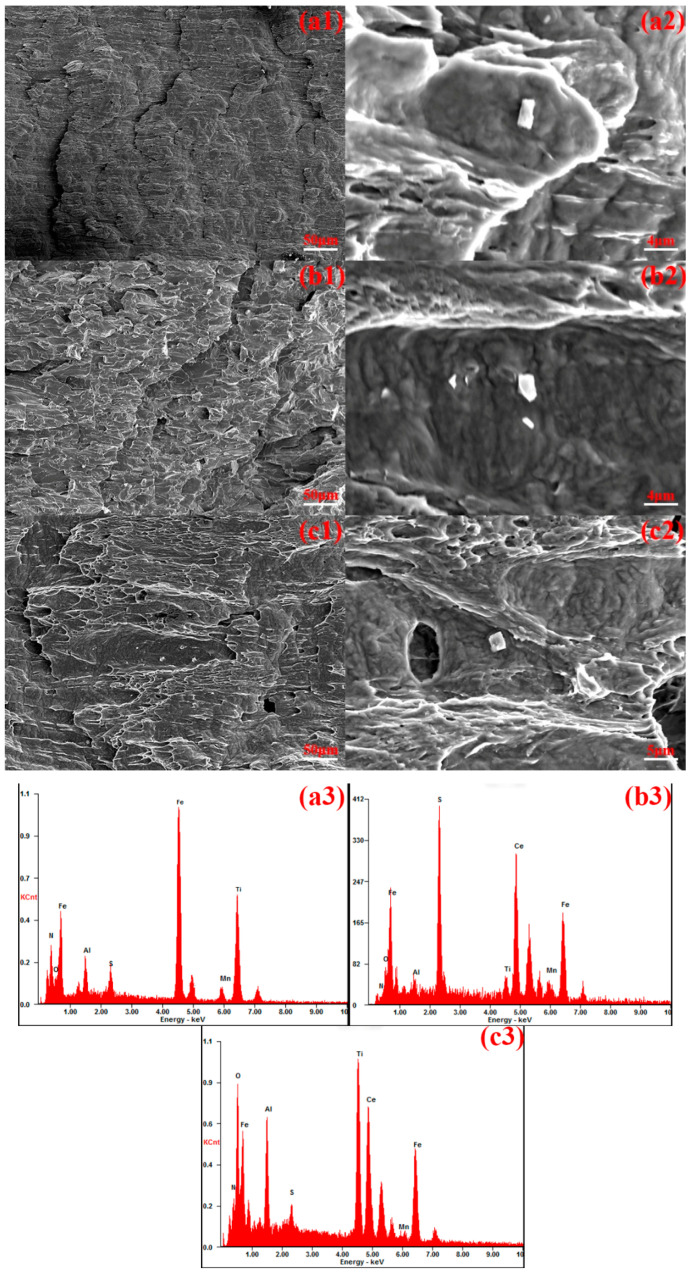
Macroscopic and microscopic morphologies and EDS spectra of impact fractures of test steels with different Ce contents, (**a1**–**a3**) 1# test steel, (**b1**–**b3**) 2# test steel, (**c1**–**c3**) 3# test steel.

Figure 6 illustrates the impact fracture surface morphology and energy spectrum analysis results for experimental steels with varying cerium (Ce) contents. As observed in Figure 6a1–c1, the fracture surface of Sample 1 exhibits uneven characteristics, with shallow and fewer tear ridges and dimples. In contrast, the fracture surface of Sample 2 displays a greater number of small river-like patterns and dimples. The fracture surface of Sample 3 exhibits numerous small dimples surrounding larger dimples, with more pronounced tear ridges and dimples at the fracture site.

As observed in Figure 6a2–c3, the inclusions at the fracture surface of Sample 1 are elongated, consisting of a composite of MnS, TiN, and Al_2_O_3_. These inclusions are elevated above the matrix surface and loosely bonded to the steel matrix, thereby disrupting the continuity of the matrix. Under the influence of external forces, the accumulation of dislocations at the inclusions and grain boundaries leads to stress concentration, which subsequently induces crack formation and reduces the impact toughness of the experimental steel. In contrast to Sample 1, the inclusions at the fracture surface of Sample 2 consist of a CeAlO_3_ + MnS + TiN composite. These inclusions exhibit a more intimate bonding with the steel matrix. The fracture surface of Sample 3 contains relatively small CeAlO_3_ + MnS + TiN rare earth composite inclusions, which are tightly adhered to the steel matrix. The matrix surface remains smooth, with little to no evidence of crack formation.

The incorporation of cerium, a rare earth element, induces significant microstructural modifications in the inclusion characteristics, particularly through the morphological transformation of elongated MnS inclusions. This metallurgical modification results in the refinement of inclusion geometry, leading to the formation of discrete, spheroidized rare earth-containing composite inclusions with reduced aspect ratios. The implemented microstructural refinement demonstrates a substantial mitigation effect on the stress concentration phenomena associated with second-phase particles. This optimization effectively enhances matrix continuity by reducing the interfacial energy between inclusions and the ferrous matrix, consequently diminishing the probability of stress-induced crack nucleation at critical microstructural sites. Furthermore, this microstructural optimization contributes to enhanced fracture resistance by impeding the advancement of microcracks through multiple energy dissipation mechanisms. The resultant improvement in crack propagation resistance, coupled with optimized grain boundary characteristics, leads to a substantial enhancement in the fracture toughness properties of the investigated steel specimens. The impact toughness of Sample 3, with a rare earth content of 0.0025 wt.%, is the highest among the tested steels.

### 3.5. Corrosion Rate Calculation

**Figure 7:**
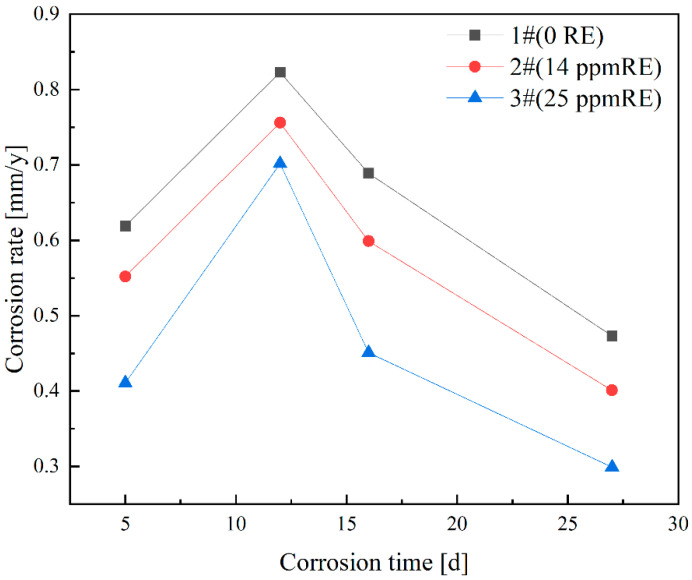
Corrosion rates of test steels with different Ce contents at different corrosion times.

To simulate a marine corrosion environment, salt spray corrosion testing was conducted on three sets of experimental steels. The experimental protocol involved subjecting the specimens to four distinct corrosion intervals, specifically 5, 12, 16, and 27 days of exposure. The degradation kinetics were quantitatively assessed through gravimetric analysis, wherein the mass reduction in the specimens was meticulously measured to determine the corrosion progression rates. The graphical representation in Figure 7 illustrates the mean corrosion kinetics of Ce-modified steel specimens across different immersion durations. The data plots demonstrate a characteristic parabolic trend, where the degradation rates of all three experimental steel variants exhibit an initial ascending phase followed by a subsequent descending pattern with prolonged exposure time. Specifically, the curve of the average corrosion rate can be divided into three distinct stages:

Stage 1: During the corrosion period of 5 to 12 days, the corrosion rates exhibit an increasing trend. The corrosion rate of Sample 1 rises from 0.619 mm/y to 0.823 mm/y, that of Sample 2 increases from 0.552 mm/y to 0.756 mm/y, and the corrosion rate of Sample 3 grows from 0.411 mm/y to 0.702 mm/y. During this stage, the corrosion products have not yet fully covered the steel substrate, resulting in a thin rust layer. This allows for increased interaction with the abundant oxygen in the environment, thereby accelerating the corrosion process.

Stage 2: During the corrosion period of 12 to 16 days, the corrosion rates begin to decline. The corrosion rate of Sample 1 decreases from 0.823 mm/y to 0.689 mm/y, that of Sample 2 drops from 0.756 mm/y to 0.599 mm/y, and the corrosion rate of Sample 3 reduces from 0.702 mm/y to 0.451 mm/y. As the corrosion time progresses, the corrosion products on the steel substrate gradually accumulate, leading to the formation of a denser rust layer that effectively covers the substrate. This development enhances the steel’s resistance to corrosion, resulting in a reduction in the corrosion rate.

Stage 3: During the corrosion period from 16 to 27 days, the corrosion rate stabilizes. The protective rust layer in all three test steels transitions from an outer layer to a denser inner layer, where alloying elements accumulate. This accumulation significantly impedes the further penetration of corrosive media, ultimately leading to a stabilization of the corrosion rate.

These experimental data reveal a significant inverse correlation between rare earth element concentration and material degradation rates. Specifically, when subjected to equivalent exposure periods, the three investigated steel specimens demonstrate progressively reduced corrosion susceptibility corresponding to incremental additions of rare earth constituents.

### 3.6. Macroscopic Corrosion Morphology Analysis

**Figure 8:**
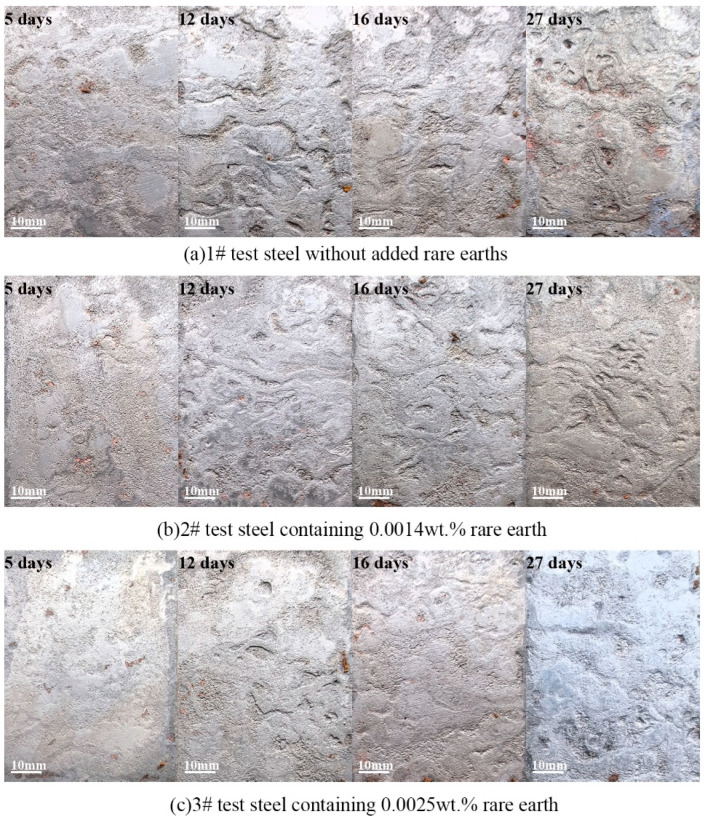
The macroscopic morphology of the test steel after 5 days, 12 days, 16 days, and 27 days of salt spray corrosion test was carried out after de-rusting, (**a**) 1# test steel, (**b**) 2# test steel, (**c**) 3# test steel.

Figure 8 presents the macro morphology of the three test steels after descaling, following the simulated marine atmospheric salt spray corrosion tests conducted at 5, 12, 16, and 27 days. After 5 days of corrosion, the substrate surfaces of all three groups of experimental steels exhibited minimal corrosion traces. After 12 days of corrosion, irregular corrosion marks appeared on the surfaces of the three test steels. These marks, formed by the interconnection of multiple pitting sites, indicate a transition from localized pitting corrosion to more uniform corrosion. Microstructural analysis of Steel Sample 1 reveals substantial surface degradation characteristics, manifested as pronounced morphological irregularities. The specimen exhibits multiple well-defined localized corrosion features, including considerable pit formation with significant depth penetration. Steel Sample 2 exhibits shallower corrosion traces. Steel Sample 3 shows the fewest corrosion pits, indicating that the rust layer on the untreated steel is loose and porous, failing to prevent chloride ion (Cl^−^) penetration effectively. However, the addition of rare earth elements significantly improved this situation. The microstructural analysis revealed that Steel Sample 1 demonstrated significant surface degradation characteristics between days 16 and 27 of the corrosion process. The substrate exhibited extensive localized corrosion phenomena, manifested as a high density of surface cavities and pronounced pit formation, indicative of advanced pitting corrosion progression. In contrast, Steel Samples 2 and 3 showed fewer instances of pit aggregation, with corrosion primarily occurring as continuous degradation.

Based on the analysis of the inclusions, it can be concluded that the steel substrate, influenced by harmful inclusions, is prone to a transition from pitting corrosion to internal corrosion. The addition of rare earth metals induced a significant modification in the morphology and distribution of non-metallic inclusions present in the steel matrix. This metallurgical refinement process effectively reduced the population density of deleterious inclusions, particularly those exhibiting unfavorable characteristics that compromise material performance. This, in turn, reduced the frequency of pitting corrosion and facilitated a shift from localized to uniform corrosion. The experimental results demonstrated a remarkable improvement in the anti-corrosion properties of the steel specimen. This enhancement in corrosion performance can be attributed to the optimized microstructural characteristics and the formation of protective surface layers, which effectively inhibited electrochemical reactions at the metal–electrolyte interface.

### 3.7. Effect of Rare Earth Ce on the Cross-Section of Rust Layer of Experimental Steel

**Figure 9:**
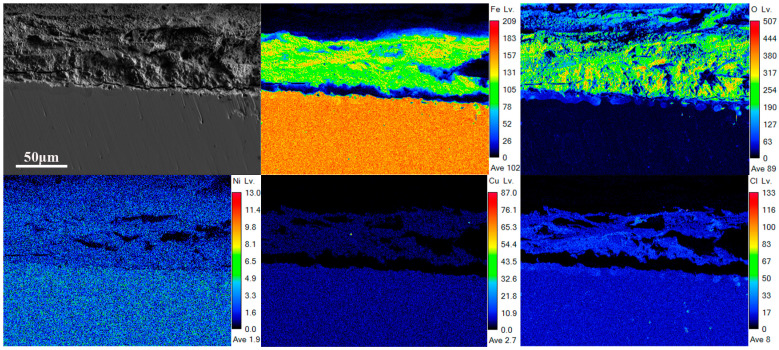
EPMA spectrum of 27d rust layer cross-section of 1# experimental steel without rare earth.

**Figure 10:**
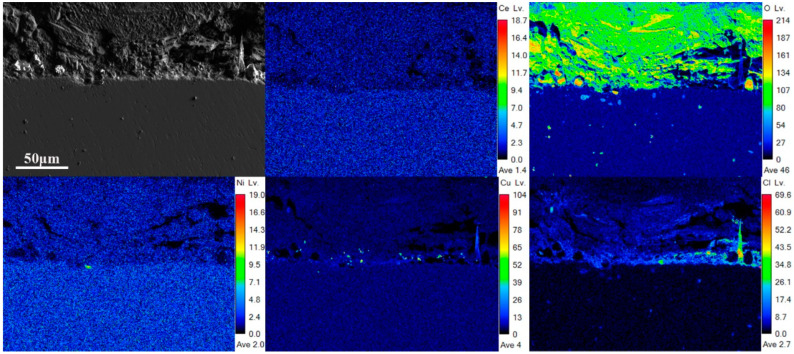
EPMA spectrum of 27d rust layer cross-section of experimental steel containing rare earth 3#.

Figure 9 and Figure 10 present the EPMA spectra of Steel Sample 1 and Steel Sample 3 after a 27-day immersion period. Microstructural characterization revealed distinctive fracture patterns within the corrosion products, as illustrated in Figure 9. The presence of discontinuous fissures at the interface between the oxide layer and metallic substrate suggests insufficient adhesion strength and structural integrity of the formed corrosion products. This morphological feature further indicates the development of a porous and mechanically unstable oxide structure on the specimen surface. The spectra of Cl and O elements reveal that the rust layer was unable to effectively prevent the penetration of corrosive ions into the steel substrate. Comparative analysis of the elemental distribution profiles demonstrates a significant alteration in the chemical composition of the corrosion products. The incorporation of rare earth elements was found to promote substantial copper accumulation in the oxide matrix. In contrast, chloride ion penetration showed no detectable concentration gradient across the corrosion layer interface, suggesting effective inhibition of Cl^−^ migration through the protective film. This suggests that the introduction of rare earth elements facilitates the accumulation of copper in the rust layer, allowing it to serve as the core for the crystallization of hydroxy iron oxide. Furthermore, the incorporation of rare earth elements promotes the refinement and densification of the rust layer’s microstructure, thereby enhancing its stability. The proposed mechanism demonstrates a significant inhibitory effect on chloride-induced degradation processes at the metal–electrolyte interface. Through the formation of a stable, protective barrier, this approach substantially decreases the electrochemical activity and mass transport kinetics, resulting in a marked reduction in corrosion propagation rates. Consequently, the modified steel exhibits superior anti-corrosion performance, as evidenced by the improved electrochemical parameters and extended service life in aggressive environments.

### 3.8. Nyquist Plot Analysis

**Figure 11:**
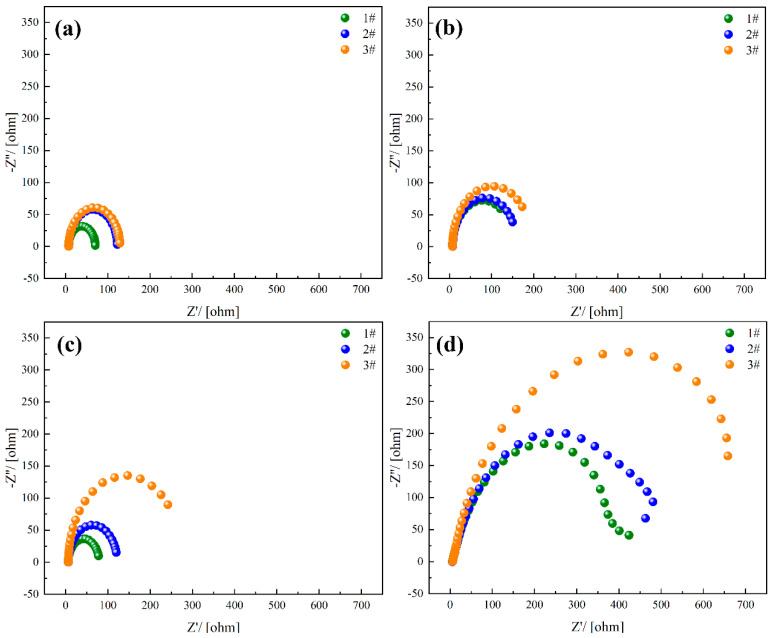
Nyquist curves of test steel soaked in 3.5% NaCl solution for different times, (**a**) 5 days, (**b**) 12 days, (**c**) 16 days, (**d**) 27 days.

**Figure 12:**
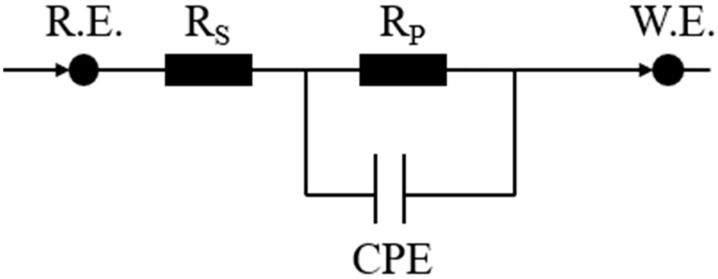
Equivalent circuit diagram of fitted data, the term “R_s_” represents the resistance of the corrosion solution; “R_p_” denotes the polarization resistance; “CPE” refers to the Constant Phase Element.

Figure 11 presents the Nyquist plots obtained from the electrochemical tests of experimental steels with varying rare earth contents, with the equivalent circuit model used for the fitting process shown in Figure 12. Electrochemical capacitance reflects the impedance characteristics of the material’s surface to electrochemical reactions. An increase in the radius of the arc typically indicates an enhancement in the material’s surface corrosion resistance. Electrochemical impedance spectroscopy analysis, as presented in Figure 11, reveals a consistent pattern in the corrosion kinetics among the three steel variants under investigation. The Nyquist plots demonstrate a progressive expansion of the semicircular region with prolonged exposure duration, indicating a systematic enhancement in the charge transfer resistance at the electrode–electrolyte interface. This temporal evolution of impedance characteristics suggests the development of increasingly protective surface films across all experimental conditions. Under the same corrosion duration, the radius of the capacitive arc increases with the rise in cerium (Ce) content, and the maximum radius is achieved when the Ce content reaches 0.0025 wt.%.

Extensive empirical investigations have demonstrated that the incorporation of lanthanide series elements into metallic matrices leads to a substantial diminution in the concentration of deleterious non-metallic inclusions, thereby effectively mitigating the propensity for localized corrosive phenomena. This leads to a more uniform electrochemical behavior in the experimental steels, manifested as an increase in electrochemical capacitance, specifically reflected by the enlargement of the capacitive arc radius. Furthermore, rare earth elements contribute to refining the grain structure and reducing microscopic defects that may initiate corrosion, effectively inhibiting the propagation of corrosion along grain boundaries. This results in a reduction in the corrosion rate in the experimental steels, further enhancing their electrochemical capacitance performance.

## 4. Conclusions

This study employs techniques such as SEM, EPMA, and Nyquist plots to investigate the effects of rare earth elements on bridge steel and their underlying mechanisms. Experimental findings demonstrate that the strategic integration of cerium (Ce) as an alloying constituent induces substantial improvements in the impact characteristics and electrochemical durability of structural steel utilized in bridge applications. In terms of inclusions, compared with the findings of Opiela [23], Liu [25], and others, the addition of Ce alone yields better results than the addition of both La and Ce. The size of non-metallic inclusions and rare earth compound inclusions decreases. The following conclusions are drawn:

(1) Rare earth elements facilitate grain refinement and enhance the distribution of pearlite. Thermodynamic calculations using FactSage software demonstrate that, upon the addition of rare earth Ce, the sequence of inclusion precipitation as the temperature decreases follows the order: CeAlO_3_ > Al_2_O_3_ > TiN > MnS. The incorporation of cerium (Ce) as a rare earth additive exerts a substantial modification effect on the morphological characteristics of non-metallic inclusions within the steel matrix, resulting in a distinct phase transformation from irregular geometries to spheroidized configurations. Furthermore, the size of the inclusions exhibits a decreasing trend, with the composition transitioning from Al_2_O_3_+TiN to CeAlO_3_+TiN rare earth composite inclusions.
(2) Comprehensive experimental data indicate a positive correlation between the concentration gradient of cerium (Ce) and the Charpy impact energy values across all investigated steel specimens. The microstructural analysis further substantiates that the introduction of rare earth constituents induces significant morphological transformations in secondary phase inclusions at fracture interfaces, resulting in enhanced crack retardation capabilities and consequent improvement in fracture toughness parameters of the tested alloys.
(3) Under the same corrosion duration, an increase in rare earth content leads to a gradual reduction in the corrosion rate of all three experimental steel groups and facilitates the transition from pitting corrosion to uniform corrosion. The incorporation of rare earth elements (REEs) facilitates the preferential accumulation of copper species within the corrosion product layer, thereby improving its structural integrity and corrosion resistance. Electrochemical impedance spectroscopy analysis revealed a positive correlation between the diameter of Nyquist semicircles and cerium concentration in REEs, indicating enhanced protective properties with increasing Ce content under identical exposure conditions.

The experimental findings reveal that the introduction of cerium (Ce) as an alloying element significantly refines the metallurgical structure of bridge steel, leading to a marked improvement in its impact performance and service performance. This modification can extend the material’s service life while also potentially reducing maintenance frequency and costs, thereby mitigating safety risks. The mechanistic understanding of cerium’s role in modifying the structural and functional characteristics of bridge-grade steel remains an area requiring comprehensive exploration through systematic research endeavors.

## Figures and Tables

**Table 1:** Chemical composition of the tested steels (wt.%).

Steel	C	Si	Mn	Ti	Cu	Ni	Al	S	P	Ce
0#	≤0.17	≤0.5	1.0–1.2	≤0.02	≤0.7	≤1.2	≥0.015	≤0.025	≤0.025	0
1#	0.017	0.431	1.051	0.025	0.581	1.014	0.004	0.021	0.027	0.0000
2#	0.010	0.453	1.049	0.051	0.603	1.019	0.021	0.018	0.022	0.0014
3#	0.014	0.451	1.042	0.054	0.615	1.015	0.018	0.019	0.016	0.0025

**Table 2:** Average grain size of test steel.

Steel	1# Test Steel	2# Test Steel	3# Test Steel
Average grain size	62.4 μm	57.3 μm	55.7 μm

## Data Availability

The original contributions presented in this study are included in the article. Further inquiries can be directed to the corresponding authors.
